# Resting Heart Rate and Incident Atrial Fibrillation in Black Adults in the Jackson Heart Study

**DOI:** 10.1001/jamanetworkopen.2024.42319

**Published:** 2024-10-30

**Authors:** Vidhushei Yogeswaran, Kerri L. Wiggins, Colleen M. Sitlani, Leonard Ilkhanoff, Emelia J. Benjamin, Susan R. Heckbert, Wondwosen Kassahun-Yimer, James S. Floyd

**Affiliations:** 1 Cardiovascular Health Research Unit, University of Washington, Seattle; 2 Department of Medicine, University of Washington, Seattle; 3 Inova Heart and Vascular Institute, Fall Church, Virginia; 4 Cardiovascular Medicine Section, Department of Medicine, Boston Medical Center, Boston University Chobanian and Avedisian School of Medicine, Boston, Massachusetts; 5 Department of Epidemiology, Boston University School of Public Health, Boston, Massachusetts; 6 Department of Epidemiology, University of Washington, Seattle; 7 Department of Data Science, University of Mississippi Medical Center, Jackson

## Abstract

**Importance:**

Resting heart rate (RHR) is a widely available measure of cardiovascular fitness that has been associated with several cardiovascular outcomes. RHR has previously been associated with the risk of atrial fibrillation (AF) among individuals of European ancestry, but little is known about this association in Black adults.

**Objective:**

To evaluate the association between RHR and incident AF in a large community-based sample of Black adults, independently of established risk factors.

**Design, Setting, and Participants:**

This cohort study uses data from the Jackson Heart Study, a prospective community-based cohort in Jackson, Mississippi. Participants without prevalent AF were included and were monitored for new-onset AF during follow-up, from 2000 through 2016. Data analysis was performed from August 1 to December 11, 2023.

**Exposure:**

RHR was assessed from resting 12-lead electrocardiograms performed at examination 1 (2000-2004) and examination 3 (2009-2013).

**Main Outcomes and Measures:**

AF was identified from study electrocardiograms, hospitalization discharge diagnosis codes, and Medicare claims diagnosis codes. Cox regression was used to evaluate the association between baseline (examination 1) RHR and incident AF, adjusting for established AF risk factors.

**Results:**

Among 4965 Black adults eligible for analysis, the mean (SD) age was 55 (13) years, 1830 (37%) were male, and the mean (SD) RHR at baseline was 65 (11) beats per minute (bpm). During a median (IQR) 14 (12-15) years of follow-up, there were 458 incident AF events, resulting in an incident rate of 7.5 per 1000 person-years (95% CI, 6.8-8.2 incidents per 1000 person-years). Each 10-bpm higher RHR was associated with a 9% higher risk of incident AF after adjustment for AF risk factors (hazard ratio, 1.09; 95% CI, 1.00-1.19). In a sensitivity analysis that excluded individuals with prior heart failure, prior myocardial infarction, and antiarrhythmic medication use at baseline, the hazard ratio was 1.14 (95% CI, 1.02-1.28). There was little evidence of effect modification of these associations by age, sex, body mass index, hypertension, or physical activity level.

**Conclusions and Relevance:**

In this large prospective cohort study of Black adults, elevated baseline RHR was associated with increased risk of incident AF, consistent with findings from previous studies of European ancestry populations. Future research should focus on determining whether RHR can be used to screen patients at high risk of AF.

## Key Points

**Question:**

Is resting heart rate (RHR) associated with new-onset atrial fibrillation (AF) in Black adults?

**Findings:**

In this cohort study of 4965 Black adults, higher RHR was associated with increased risk of new-onset AF during a median of 14 years of follow-up after adjustment for established AF risk factors. Every 10 beats per minute increase in RHR was associated with a 9% increased risk of AF.

**Meaning:**

These findings suggest that RHR is associated with new-onset AF in Black adults, independently of other established risk factors; additional research is needed to determine whether RHR can improve the selection of individuals for AF screening, which may help to mitigate long-standing health disparities.

## Teaser

This cohort study examines the association between resting heart rate and new-onset atrial fibrillation in a large community-based sample of Black adults in Jackson, Mississippi.

## Introduction

Atrial fibrillation (AF), the most common sustained cardiac arrhythmia, can lead to heart failure, stroke, dementia, and death.^1^ Large population-based studies have reported that the incidence of clinically recognized AF is lower in Black adults than in White adults^2,3^; however, cross-sectional studies using unbiased methods of arrhythmia detection suggest that the prevalence rates are similar.^4^ Paradoxically, Black adults with AF have higher rates of ischemic stroke and AF-related complications compared with White adults.^5,6^ Given these disparities, there is a need to improve AF screening and detection for Black adults.

Resting heart rate (RHR) is a readily available measurement of physical and cardiovascular fitness^7^ that is associated with increased risk of some cardiovascular outcomes and mortality.^8,9^ Less is known about its association with AF, given inconsistent results from prior studies^10,11,12^ that included few Black participants. We conducted a longitudinal analysis in the Jackson Heart Study (JHS), the largest prospective cohort study of cardiovascular diseases in Black adults, to determine whether RHR is associated with increased risk of AF in this population.

## Methods

JHS is a prospective cohort study that recruited 5306 self-identified Black adults aged 20 to 95 years from a tri-county area around Jackson, Mississippi. The first field center examination (2000-2004; examination 1) included questionnaires on medical and behavioral health, anthropometry, electrocardiography (ECG), echocardiography, and biomarker assays.^13^ Additional field center examinations were conducted in 2005 to 2008 (examination 2) and 2009 to 2013 (examination 3). AF was ascertained through 2016 from study ECGs performed at examination 1 and examination 3, from hospitalization diagnosis codes, and from Medicare claims.^13^ All participants provided written informed consent, and the study protocols were approved by local institutional review boards. This study followed the Strengthening the Reporting of Observational Studies in Epidemiology (STROBE) reporting guidelines.

### Resting Heart Rate

RHR was assessed from a supine 10-second resting 12-lead ECG at examination 1 and examination 3 recorded using a Marquette MAC/PC ECG recorder (Marquette Electronics). The ECG was obtained after the electrodes were placed on the skin for at least 2 to 3 minutes.

### Covariates

Covariates assessed at examination 1 included age, sex, height, weight, systolic blood pressure, diastolic blood pressure, antihypertensive medication use, current smoking, prevalent diabetes, and self-reported history of myocardial infarction (MI) and heart failure (HF). Prevalent diabetes was defined as self-reported diabetes, diabetes medication use, hemoglobin A_1c_ greater than or equal to 6.5% (to convert to proportion of total hemoglobin, multiply by 0.01), or fasting blood glucose greater than or equal to 126 mg/dL (to convert to millimoles per liter, multiply by 0.0555). ECG variables (PR interval and left ventricular hypertrophy by Cornell criteria), echocardiography variables (left ventricular mass, left ventricular ejection fraction, and left atrial maximal internal diameter), and laboratory values were also assessed at examination 1.

### Statistical Analysis

Statistical analysis was performed from August 1 to December 11, 2023. Data were analyzed using Stata statistical software version 13 (StataCorp). Participants with prevalent AF at examination 1 (264 participants), a ventricular paced rhythm on examination 1 or examination 3 ECG (7 participants), no baseline ECG (18 participants), no palpated RHR at examination 1 (18 participants), or a PR duration of 0 (34 participants) were excluded from the analysis. We evaluated associations between RHR and incident AF with Cox regression. The association between RHR as a linear term and as a nonlinear (cubic spline) term was evaluated using likelihood ratio testing, which was found to be nonsignificant. Statistical significance was set at 2-sided *P* < .05.

In the primary analysis, RHR was modeled as a linear term, adjusting for age, sex, height, weight, systolic blood pressure, diastolic blood pressure, antihypertensive medication use, current smoking, prevalent diabetes, prior MI, and prior HF as AF risk factors. We conducted additional analyses that modeled RHR as a time-varying exposure measured at both examination 1 and examination 3, and performed sensitivity analyses additionally adjusted for ECG, echocardiographic, and laboratory risk factors and separately excluded participants with prevalent HF or MI or who used digoxin, antiarrhythmic medications, or negative chronotropic medications. We also evaluated for effect modification by age, sex, body mass index (calculated as weight in kilograms divided by height in meters squared), hypertension status, and moderate-to-vigorous physical activity (MVPA) levels by testing interactions between RHR and these patient factors (eTable 1 in Supplement 1). The presence of MVPA was assessed through the JHS Activity Survey, where sports and/or exercise activities were converted into minutes per week. In our secondary analysis, we evaluated whether change in RHR from examination 1 to examination 3, adjusted for examination 1 RHR and covariates, was associated with incident AF. In this analysis, we excluded anyone with incident AF on or prior to visit 3. Multiple imputation with chained equations was used for missing data.^14^

## Results

Among 4965 eligible participants, the mean (SD) age was 55 (13) years, 37% (1830 participants) were male, and 50% (2463 participants) used antihypertensive medications (Table 1). The mean (SD) baseline RHR was 65 (11) beats per minute (bpm), and during a median (IQR) 14 (12-15) years of follow-up, there were 458 incident AF events, resulting in an incident rate of 7.5 per 1000 person-years (95% CI, 6.8-8.2 incidents per 1000 person-years).

**Table 1.** Baseline Characteristics of the Study Population

Characteristic	Participants, No. (%) (N = 4965)	Missing data, No. (%)
Age, mean (SD), y	55 (13)	NA
Age ≥60 y	1918 (39)	NA
Sex		
Female	3135 (63)	NA
Male	1830 (37)	NA
Weight, mean (SD), kg	91 (22)	2 (0.04)
Height, mean (SD), cm		
Female participants	164 (6)	2 (0.04)
Male participants	177 (7)	
Systolic blood pressure, mean (SD), mm Hg	127 (17)	1 (0.02)
Diastolic blood pressure, mean (SD), mm Hg	76 (9)	1 (0.02)
Antihypertensive medication use	2463 (50)	234 (4.7)
Current smoking	649 (13)	43 (0.9)
Prevalent diabetes	1074 (22)	45 (0.9)
Prior heart failure	133 (3)	206 (4.2)
Prior myocardial infarction	265 (5)	NA
Electrocardiogram results		
Left ventricular hypertrophy by Cornell Criteria	492 (10)	NA
Maximal PR interval, mean (SD), ms	179 (28)	82 (1.7)
Laboratory results		
Estimated glomerular filtration rate, mean (SD), mL/min/1.73 m^2^	95 (22)	62 (1.2)
Brain-type natriuretic peptide, mean (SD), pg/mL	18 (47)	996 (20)
Echocardiographic measures		
Left atrial maximal diameter mean (SD), mm	35 (4)	254 (5.1)
Left ventricular mass, mean (SD), g	149 (52)	248 (5.0)
Left ventricular ejection fraction	62 (8)	233 (4.7)
Medications		
Negative chronotropic medication use	855 (17)	323 (6.5)
Digoxin use	54 (1)	362 (7.3)
Antiarrhythmic medication use	7 (0.1)	368 (7.4)

Abbreviation: NA, not applicable.

SI conversion factor: To convert brain-type natriuretic peptide to nanograms per liter, multiply by 1.

In the primary analysis adjusted for AF risk factors, each 10-bpm higher baseline RHR was associated with a hazard ratio (HR) for incident AF of 1.09 (95% CI, 1.00-1.19) (Table 2). Excluding participants with cardiovascular disease or antiarrhythmic medication use at baseline (HR, 1.14; 95% CI, 1.02-1.28), modeling RHR as a time-varying exposure (HR, 1.09; 95% CI, 1.01-1.19), and adjusting for additional ECG and echocardiographic risk factors (HR, 1.13; 95% CI, 1.04-1.24) resulted in larger HRs.

**Table 2.** Association Between RHR as a Continuous Parameter and Incident Atrial Fibrillation

Variable	No. of patients	No. of events	Model 1^a^		Model 2^b^	
			HR (95% CI)	*P* value	HR (95% CI)	*P* value
Baseline RHR	4965	458	1.09 (1.00-1.19)	.047	1.13 (1.04-1.24)	.01
Time-varying RHR	4965	458	1.09 (1.01-1.19)	.03	1.12 (1.03-1.22)	.01
Baseline RHR with prior heart failure, prior myocardial infarction, and antiarrhythmic medicine use excluded	3912	263	1.14 (1.02-1.28)	.02	1.23 (1.09-1.38)	.001

Abbreviations: HR, hazard ratio; RHR, resting heart rate.

^a^ Model 1 was adjusted for baseline covariates, including age, sex, height, weight, systolic blood pressure, diastolic blood pressure, antihypertensive medication use, current smoking, prevalent diabetes, prior heart failure, and prior myocardial infarction.

^b^ Model 2 was adjusted for model 1 covariates plus estimated glomerular filtration rate, brain-type natriuretic peptide, left ventricular hypertrophy, PR interval, left atrial maximal internal diameter, left ventricular mass, and left ventricular ejection fraction.

There was no evidence of effect modification by age, sex, hypertension status, body mass index, or MVPA (eTable 1 in Supplement 1). Evaluating change in RHR from examination 1 to examination 3 as the exposure of interest and adjusting for the baseline RHR, there was no significant association with incident AF (HR, 1.13; 95% CI, 0.96-1.32) (eTable 2 in Supplement 1).

## Discussion

In this prospective cohort study of Black adults, higher RHR was associated with greater risk of incident AF. Each 10-bpm higher RHR was associated with a 9% increased risk of incident AF.

In prior studies^10,11,12^ of mostly European ancestry individuals, the association between RHR and AF has been inconsistent. In the Atherosclerosis Risk in Communities study, which included 24% Black adults, a baseline RHR greater than 80 bpm was associated with a greater risk of incident AF compared with an RHR of 55 to 79 bpm.^10^ Notably, there was no evidence of effect modification by race.^10^ In the Copenhagen Electrocardiographic Study, both high and low RHR were associated with an increased risk of AF.^15^ In our study, individuals with the greatest RHR had the highest risk of incident AF, and there was no evidence of a nonlinear or U-shaped association.

### Strengths and Limitations

In this largest analysis of RHR as a risk factor for AF in Black adults, strengths include the standardized assessment of RHR and of AF risk factors, and the broad longitudinal surveillance for AF events from multiple data sources. There are also several limitations. RHR was assessed at only 2 time points and does not capture short-term variability in RHR. AF is often subclinical, and our reliance on health care encounters to identify AF events reflects only clinically recognized AF. Although we did not identify a U-shaped association between RHR and AF risk, we may have lacked power to identify a small increase in risk among individuals with the lowest RHR. Furthermore, despite the high-quality assessment of AF risk factors, residual confounding is a possible explanation for our findings.

## Conclusions

We found that RHR, a simple marker of cardiorespiratory fitness and autonomic tone, was associated with increased risk of AF in Black adults independently of established AF risk factors, and individuals with the highest RHR had the greatest risk of incident AF. Our study underscores the importance of RHR as a measure to estimate the risk of AF in Black adults and the importance of conducting investigations in diverse populations. Additional research is needed to determine whether RHR can help to improve the selection of individuals for AF screening, which may help to mitigate long-standing health disparities.

## Notes

Supplement 1. **eTable 1.** Associations between RHR and incident AF overall and in subgroups of participants **eTable 2.** Associations between change in RHR and incident AF

Supplement 2.
Data Sharing Statement
