# Supplementary material for: Resting Heart Rate and Incident Atrial Fibrillation in Black Adults in the Jackson Heart Study
Source: JAMA Netw Open. 2024 Oct 30;7(10):e2442319. doi: 10.1001/jamanetworkopen.2024.42319 (PMC11525598; doi:10.1001/jamanetworkopen.2024.42319)
Supplement: Supplement 1. — eTable 1. Associations between RHR and incident AF overall and in subgroups of participants eTable 2. Associations between change in RHR and incident AF [file jamanetwopen-e2442319-s001.pdf]

Supplemental Online Content

Yogeswaran V, Wiggins KL, Sitlani CM, et al. Resting heart rate and incident atrial fibrillation in Black adults in the Jackson Heart Study. *JAMA Netw Open*. 2024;7(10):e2442319. doi:10.1001/jamanetworkopen.2024.42319

eTable 1. Associations between RHR and incident AF overall and in subgroups of participants

eTable 2. Associations between change in RHR and incident AF

This supplemental material has been provided by the authors to give readers additional information about their work.

© 2024 Yogeswaran V et al. *JAMA Network Open.*

eTable 1: Associations between RHR and incident AF overall and in subgroups of participants

Variable	Total N*	Hazard Ratio (95% CI)	Interaction P-Value
Sex			0.82
Female	3135	1.09 (0.98-1.22)	
Male	1830	1.09 (0.95-1.24)	
Age (continuous), years	4965	0.99 (0.98-1.00)	0.06
Hypertension status*			0.44
No-hypertension	2025	1.23 (0.96-1.56)	
Hypertensive	2940	1.07 (0.98-1.18)	
BMI (continuous), kg/m^2	4965	1.01 (0.99-1.02)	0.37
Moderate/vigorous physical activity*			0.24
No	2431	1.04 (0.93-1.17)	
Yes	2534	1.15 (1.01-1.32)	

The model was adjusted for baseline variables: age, sex, height, weight, SBP, DBP, antihypertensive medication use, current smoking, prevalent diabetes, prior heart failure, and prior myocardial infarction. Hypertension was defined as SBP ≥ 140 mmHg, DBP ≥ 90 mmHg, or the use of antihypertensive medications. Moderate/vigorous physical activity was calculated as the presence of any (≥ 0) calculated minutes per week of moderate or vigorous physical activity. Effect modification was assessed by testing the interaction of RHR multiplied by the adjustment variables.

© 2024 Yogeswaran V et al. *JAMA Network Open.*

eTable 2: Associations between change in RHR and incident AF

	N		Model 1*		Model 2**	
	Total	Events	HR (95% CI)	p-value	HR (95% CI)	p-value
Change in RHR	3309	163	1.13 (0.96-1.32)	0.14	1.13 (0.96-1.32)	0.13
Change in RHR with prior heart failure, prior myocardial infarction, and antiarrhythmic med use excluded	2845	156	1.02 (0.82-1.26)	0.88	1.02 (0.83-1.26)	0.83

*Model 1 was adjusted for baseline variates: age, sex, height, weight, SBP, DBP, antihypertensive medication use, current smoking, prevalent diabetes, prior heart failure, and prior myocardial infarction. **Model 2 was adjusted for Model 1 covariates plus eGFR, BNP, LVH, PR interval, left atrial maximal internal diameter, left ventricular mass, and left ventricular ejection fraction.

© 2024 Yogeswaran V et al. *JAMA Network Open.*
