# Supplementary material for: Resting Heart Rate and Incident Atrial Fibrillation in Black Adults in the Jackson Heart Study
Source: JAMA Netw Open. 2024 Oct 30;7(10):e2442319. doi: 10.1001/jamanetworkopen.2024.42319 (PMC11525598; doi:10.1001/jamanetworkopen.2024.42319)
Supplement: Supplement 2. — Data Sharing Statement [file jamanetwopen-e2442319-s002.pdf]

Data Sharing Statement

Yogeswaran. Resting Heart Rate and Incident Atrial Fibrillation in Black Adults in the Jackson Heart Study. *JAMA Netw Open*. Published October 30, 2024.
doi:10.1001/jamanetworkopen.2024.42319

Data
Data available: No

Additional Information
Explanation for why data not available: It is available to other researchers who submit an application to the Jackson Heart Study.
